# Oral health care in older people in long-term care facilities: An updated systematic review and meta-analyses of implementation strategies

**DOI:** 10.1016/j.ijnsa.2024.100289

**Published:** 2024-12-31

**Authors:** Lina F. Weening-Verbree, Anouk Douma, Cees P. van der Schans, Getty J. Huisman-de Waal, Annemarie A. Schuller, Sytse U. Zuidema, Wim P. Krijnen, Johannes S.M. Hobbelen

**Affiliations:** a Research Group Healthy Ageing, Allied Health Care and Nursing and FAITH Research, Hanze University of Applied Sciences, Petrus Driessenstraat 3, 9714, CA, Groningen, The Netherlands; b Center for Dentistry and Oral Hygiene, University Medical Center Groningen A, Deusinglaan 1 FB 21, 9713, AV, Groningen, The Netherlands; c Hanze University of Applied Sciences, Petrus Driessenstraat 3, 9714, CA, Groningen, The Netherlands; d Department Health Psychology, University Medical Centre Groningen, Groningen, The Netherlands; e Department Rehabilitation Medicine, University Medical Centre Groningen, Groningen, The Netherlands; f Department of IQ Healthcare, Radboud University Nijmegen Medical Center, Kapittelweg 54, 6525, EP, Nijmegen, The Netherlands; g TNO the Netherlands Organisation for applied scientific research, Sylviusweg 71, 2333, BE Leiden, The Netherlands; h Department of Primary and Long-term Care, University of Groningen, University Medical Center Groningen, P.O. Box 196 FA21, 9700, AD, Groningen, The Netherlands; i Faculty of Science and Engineering, University of Groningen, Groningen, the Netherlands

**Keywords:** Implementation, Nursing staff, Meta-analyses, Oral health, Older people, Systematic review

## Abstract

**Introduction:**

Oral health care of older people in long-term care facilities is insufficient, stressing the need for clear evidence-based implementation strategies to improve oral care. In 2013, a systematic review was performed and new evidence was published. This study aimed to gain insights into implementation strategies used to promote or improve oral health care for older people in long-term care facilities, explore their effectiveness and uncover strategy content in behavioral change techniques, and report the differences between the current results and those of the 2013 study.

**Methods:**

A systematic review of the literature according to PRISMA guidelines and meta-analyses of implementation strategies were performed. Cochrane Library, PubMed, and CINAHL databases were searched for papers published between 2011 and 2023. Strategies were identified using the Coding Manual for Behavioral Change Techniques. Meta-analyses of oral health outcomes (“plaque” and “denture plaque”) were performed with random-effects models using R language for statistical computing.

**Results:**

16 studies were included in the current results; 20 studies were included in the 2013 findings. More high-quality studies (67 %) were included in this review than in 2013 (47 %). Dental care professionals were involved in 14 of the 16 studies. Fourteen of the 16 studies used and/ or combined five or more different implementation strategies: knowledge, intention, awareness, self-efficacy, attitude, and facilitation of behavior. Implementation positively affected the knowledge and attitudes of the nursing staff; however, the oral health of older people did not necessarily improve. In the 2013 review, more studies indicated combined oral health measurements were effective (71 %) than in the current review (20 %–33 %). Meta-analysis of four studies on dental plaque (0—3 scale) showed a significant, statistically small mean difference of -.21 (CI -.36; -.07, Cohen's *d -*.29) between the control and treatment group. Meta-analysis of three studies on denture plaque (0—4 scale), showed a significant, statistically large mean difference of -.76 (CI -1.48; -.05, Cohen's *d* -.88).

**Conclusions:**

In this review, more implementation strategies and combinations were used to implement oral care in long-term care. Implementation strategies positively affected the knowledge and attitudes of nursing staff; however, the oral health of older people did not necessarily improve. Meta-analyses on plaque showed that oral care implementations are effective; for denture plaque, the effect size was large and thus may have more clinical value than for dental plaque.

**What is already known**

• Poor oral health and hygiene may contribute to adverse health outcomes such as malnutrition, pneumonia, diabetes, pain, and a decline in the well-being of older people.
• The oral hygiene and oral health of older people in long-term care facilities are insufficient.
• Although oral care is a part of fundamental nursing care, barriers to oral care are present, leading to incomplete provision of oral care for older people.

Alt-text: Unlabelled box

**What this paper adds**

• Strategies to improve oral care included knowledge, intention, awareness, self-efficacy, attitude, and facilitation of behavior, or a combination of these.
• All strategies had a statistically significant positive effect on the knowledge and attitudes of the nursing staff; however, most strategies did not consistently show a significantly positive effect on the oral health of older people.
• The implementation of oral care aimed at reducing plaque levels in older people showed more pronounced positive results for denture plaque than for dental plaque.

Alt-text: Unlabelled box

## 1 Introduction

Oral health is important, and multiple associations between oral and general health have been found: oral inflammation may disturb HbA1C levels in patients with diabetes and may contribute to rheumatoid arthritis, and both malnutrition and aspiration pneumonia are associated with poor oral health (D'Aiuto et al., 2018; Huppertz et al., 2017; Johansson, L. et al., 2016; Maarel-Wierink et al., 2013). Oral health and hygiene also contribute to social well-being and self-esteem (Castrejón-Pérez and Borges-Yáñez, 2014; Masood et al., 2017).

However, the oral health and hygiene of older people is at risk because of functional decline, loss of motor skills, polypharmacy, chronic diseases, and/or cognitive impairment (Gao et al., 2020; Janssens, Barbara et al., 2017; Lee et al., 2020; van der Putten and de Baat, 2023). Therefore, older people are more often dependent on nursing staff for “activities of daily living” (ADL), such as daily tooth brushing, which affect their maintenance of oral health (Saintrain et al., 2018; Tuuliainen et al., 2020).

The provision of oral healthcare for older people in long-term care facilities is frequently inadequate and does not adhere to established guidelines (Hoben et al., 2017; Weening-Verbree, et al., 2021; Yoon et al., 2018). Various studies have attributed this deficiency to several factors, including time constraints, non-compliant older residents, insufficient supplies, lack of knowledge regarding proper oral care techniques, and inadequate collaboration between dental and nursing staff (Gopalakrishnan et al., 2019; Göstemeyer et al., 2019; Hoben et al., 2017). Despite being an integral component of fundamental care, oral healthcare is often categorized as “missed nursing care” (Edfeldt et al., 2023; Mainz et al., 2024). The high workload and understaffing prevalent in these facilities further contribute to incomplete or neglected provision of oral care. Consequently, there is a pressing need for evidence-based implementation strategies to enhance oral healthcare, emphasizing its role as essential nursing care.

Implementation strategies often used to improve oral care in nursing homes include knowledge, self-efficacy, and facilitation of behavior (Weening-Verbree, L. et al., 2013). This was mostly operationalized as an educational meeting or presentation, training of oral care skills for nursing staff, and the supply of materials for oral care (Weening-Verbree, L. et al., 2013). Other strategies evaluated in a systematic review in 2013 addressed ”increasing memory,” “providing feedback on clinical outcomes,” and “mobilizing social norms” (Weening-Verbree, L. et al., 2013). These strategies were not often used, but were promising (Weening-Verbree, L. et al., 2013). The effects of oral care implementation in long-term nursing care facilities could not be attributed to one or more implementation strategies.

Oral health care for older people in nursing homes is insufficient. Although oral care programs have been implemented, these programs may not have resulted in improved oral care (Hoben et al., 2017; Hoeksema et al., 2017; Yoon et al., 2018). Since 2013, additional studies have been conducted; therefore, it is useful to report the new evidence and perform additional systematic literature reviews. Furthermore, the study results can be compared with those of the 2013 review and meta-analyses can be performed if sufficient data are available.

This study aimed to gain insights into implementation strategies used to promote or improve oral health care for older people in long-term care facilities and to explore their effectiveness, uncover strategy content in behavioral change techniques, report differences in strategies used and effectiveness between the results of the two reviews, and preferably perform a meta-analysis.

## 2 Methods

### 2.1 Search Strategy

First, the digital databases of the Cochrane Library, PubMed, and CINAHL were searched for articles published from September 2011 to June 2023, according to Weening-Verbree et al. (2013). The same MeSH terms and combinations of terms used in 2013 were applied: nursing, nursing care, geriatric nursing, nursing homes, nursing home personnel, caregivers, oral hygiene, oral health, health education, dental, and aged 80 and over (Supplementary Appendix A).

### 2.2 Procedure

After duplicates were excluded, two reviewers (LWV and AD) screened all abstracts and titles using the inclusion and exclusion criteria. Full-text papers were subjected to the same evaluation strategy by LWV and AD. Quality assessment and data extraction were performed by LWV and AD. A third reviewer (AS) was available to reach consensus in a few cases.

### 2.3 Selection criteria

Studies had to include an outcome comparison with a randomized or non-randomized comparison group or a comparison with baseline data in the case of an uncontrolled before-after design. Other inclusion criteria were:

- Population: healthcare personnel (e.g., nurses or nurse assistants) in nursing homes who were involved in the implementation, and/or older people in nursing homes or residential care facilities

- Outcome: oral health (plaque, gingivitis, or candidoses) or knowledge and beliefs of healthcare personnel

Exclusion criteria:

- studies focusing solely on the effects of drugs or oral health care products
- (nonsystematic) reviews, although their reference lists were checked for possible missed studies
- studies with three or fewer points out of seven on the quality ratings (Anderson and Sharpe, 1991). Studies that were rated three points but failed to have a positive score for “instruments used” or studies that lacked statistical analysis were also excluded.

The PRISMA guidelines were used to report the selection process of the studies (Higgins et al., 2023; Moher et al., 2009).

### 2.4 Quality assessment

The quality of the studies was assessed using a rating system adapted from Anderson and Sharpe (see Appendix B)(Anderson and Sharpe, 1991). This rating system consists of six items on the methodology of the study, including design, power, validity, and reliability of the measurement of outcomes. Items could be scored from zero to two points; score zero indicates “not present,” score one indicates “present.” The item “outcome” could be rated at two points, resulting in a total quality score per study ranging from zero to seven points. Studies that scored three–five points were graded as moderate quality, and those with six or seven points were graded as high quality, in accordance with the 2013 systematic review (Weening-Verbree, L. et al., 2013).

### 2.5 Data extraction

The content of the included studies was examined in two steps as described in 2013. First, we extracted the study characteristics using the EPOC Data Collection Checklist and Data Abstraction Form (Higgins et al., 2023), which included study objectives, setting, study design, target population, outcome measures, and descriptions of the intervention, analysis, and results. Second, we extracted all information on the content of the implementation strategy from the studies and classified the different elements using the Coding Manual for Behavioral Change Techniques (BCT) (De Bruin et al., 2009). This coding manual is a further developed and adapted version of the coding manual by Abraham and Michie for use in patient care (Abraham and Michie, 2008). It groups behavior-change techniques according to relevant behavioral determinants. Nine main categories of determinants were distinguished: knowledge, awareness, social influence, attitude, self-efficacy, intention, action control, maintenance, and facilitation of behavior (Supplementary Appendix C).

### 2.6 Data analysis

First, a descriptive analysis was performed on the implementation strategies included in the current review and the frequency with which the behavioral determinants were addressed as reported by these strategies. Second, after coding the strategy content, we analyzed the effectiveness at the level of specific strategies in the included studies. We used the presence of a statistically significant positive effect as a measure of strategic effectiveness. The effectiveness of these strategies was determined by comparing the number of studies that demonstrated effectiveness with the total number of studies using these strategies. As most studies likely addressed more than one determinant in their implementation strategies, we could only report evidence for strategies used in combination with other strategies. Third, we also examined frequently used combinations of determinants within one strategy but limited this to combinations used in more than three different studies.

An example of a frequently used combination within one strategy was an implementation strategy consisting of a theoretical lecture on oral health combined with hands-on training in toothbrushing techniques, discussion sessions, and the provision of electric toothbrushes. These strategies were coded as addressing the determinants of knowledge, self-efficacy, and facilitation of behavior.

The results of the quality assessment, strategies used, and their effectiveness are reported in the tables to provide insights. The results of the 2013 systematic review are presented along with the current results to facilitate comparison and reporting. In the analysis of the current studies, we chose to uncover strategies only among the nine main strategies to avoid repeating the results. The fourth and final step was to determine whether conducting a meta-analysis was feasible for one or more outcome measurements. Meta-analysis was performed if more than two studies could be included. Meta-analyses were conducted on two outcomes, dental plaque and denture plaque, among nursing home residents. Dental plaque was measured using the Silness and Loë plaque index, (Silness and Loe, 1964) and denture plaque was measured using the Augsburger and Elahi denture plaque index (Augsburger and Elahi, 1982). Both indices are ordinal in nature, using zero–three or zero–four scales. Higher scores indicated more severe plaque and poorer oral hygiene. Only studies using a pre-post-test RCT design using the same outcome measurement instrument and a similar follow-up time were included to interpret the meta-analysis results directly according to the mean differences. The analyses were performed using R language for statistical computing (R Core Team and R Foundation for Statistical Computing, 2021; Schwarzer et al., 2015). The baseline data of the intervention and control groups were pooled and compared to ensure randomization. The mean differences with their associated 95 % confidence intervals, Cohen's *d* and *p-*values were calculated for both dental and denture plaque. We considered *p-*values < 0.05 significant; a Cohen's *d* of .2 was considered “small” effect size, .5 “medium” effect size, and an effect size of .8 or over was considered “large”(Cohen, 1988). The mean differences were calculated using the random-effects model because we expected the effect of treatment to be similar across studies, but not identical due to random fluctuations.

## 3 Results

**Fig. 1:**
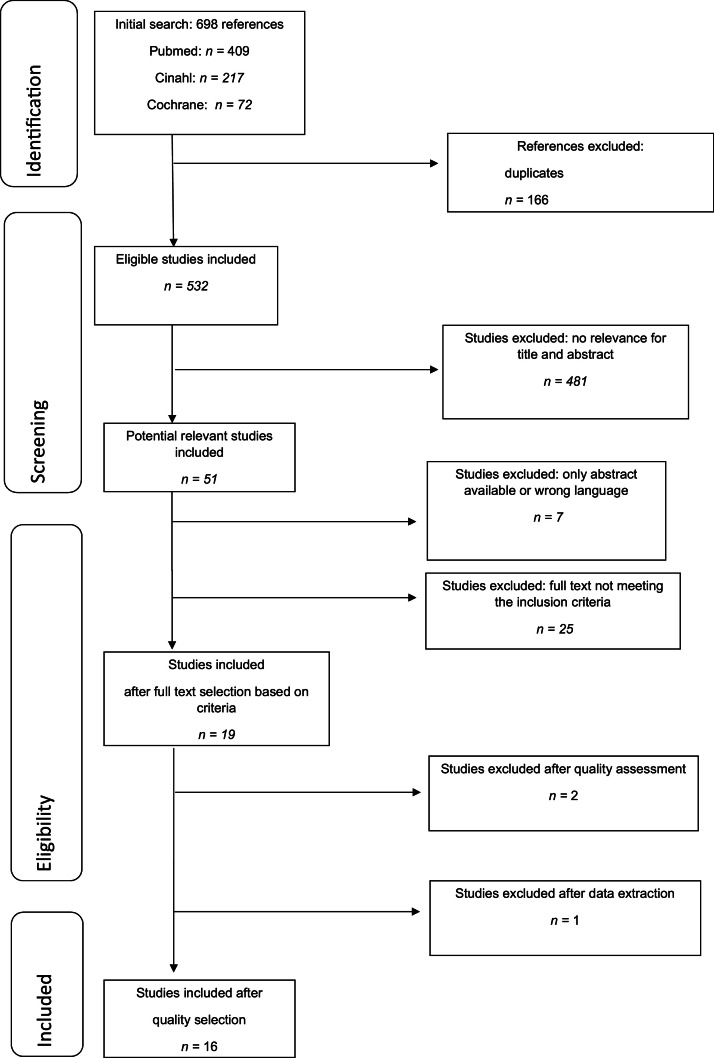
PRISMA flow diagram of the selection procedure.

The literature search resulted in 532 hits after the exclusion of duplicates. Based on titles and abstracts, 51 studies were selected for full-text assessment. After full-text reading, 19 studies met the inclusion criteria, and their quality was assessed. Fig. 1 shows a PRISMA flowchart of the data selection process. Reasons for exclusion after full-text reading were as follows: effect measurements not meeting the inclusion criteria (e.g., only microbiology), targeted intervention population not specified, and intervention not targeting nursing staff (e.g., intervention was professional dental care by dental care professionals). Two studies were excluded after the quality assessment. One study (26) was removed from the current analysis, as we discovered that this study was already included in the 2013 review; however, after acceptance in 2010, the study report was slightly adjusted, including the data of the publication date (De Visschere et al., 2012). This adjustment did not affect the results. Ultimately, the report of the current systematic review was based on 16 included studies.

### 3.1 Quality of the studies

The rating of study quality resulted in 12 high-quality studies and four moderate-quality studies, as shown in Table 1. Two studies were excluded because the statistical analysis was not clearly reported (no *p*-values or confidence intervals were given) or because of low study quality (McConnell et al., 2018; Volk et al., 2020). Among the most common quality limitations was the “lack of sample size calculation,” which was reported in only four studies (Janssens, et al., 2018; Le et al., 2012; Overgaard et al., 2022a; Putten et al., 2013). Additionally, the absence of a control group in study design was identified in seven studies (Bonwell et al., 2014; Forsell et al., 2011; McConnell et al., 2018; Portella et al., 2015; Red and O'Neal, 2020; Sloane et al., 2013; Volk et al., 2020). More detailed information on the quality ratings of the included studies can be found in Appendix D. All the studies clearly described the intervention or implemented program. As shown in Table 1, the 67 % of the studies included in this review were high quality.

**Table 1:** Quality assessment for all studies of 2024 (*n* = 18) and 2013 (*n* = 21).

Included in 2024 Author, year	Quality rating*	Included in 2013 Author, year	Quality rating*
Amerine et al., 2014	6	Boczko et al., 2009	5
Bonwell et al., 2014	4	Budtz et al., 2000	6
DeVisschere et al., 2011	6	De Visschere et al., 2012	6
Forsell et al., 2011	4	Fallon et al., 2006	6
Janssens et al., 2018	6	Frenkel et al., 2001	7
Johansson et al., 2020	6	Frenkel et al., 2002	6
Le et al., 2012	7	Isaksson et al., 2000	6
McConnell et al., 2018	3**	Jäger et al., 2009	5
Overgaard et al., 2022b	7	Kullberg et al., 2010	5
Portella et al., 2015	5	MacEntee et al., 2007	7
Red and O'Neal, 2020	5	Mojon et al., 1998	6
Schwindling et al., 2018	6	Nicol et al., 2005	6
Seleskog et al., 2018	6	Paulsson et al., 1998	5
Sloane et al., 2013	6	Paulsson et al., 2001	5
Van der Putten, 2012	7	Pronych et al., 2010	4
Volk et al., 2020	2**	Reed et al., 2006	4
Weintraub et al., 2018	6	Rivett, 2006	3**
Zenthöfer et al., 2016	6	Samson et al., 2009	5
		Simons et al., 2000	6
		Wårdh et al., 2002a	5
		Wårdh et al., 2002b	5

* 3-5 moderate quality; 6-7 high quality

** this study was excluded because no statistical analysis was described.

### 3.2 General characteristics of studies

Nine studies were randomized controlled trials (Amerine et al., 2014; De Visschere et al., 2011; Johansson et al., 2020; Le et al., 2012; Overgaard et al., 2022a; Putten et al., 2013; Seleskog et al., 2018; Weintraub et al., 2018; Zenthöfer et al., 2016), two were controlled clinical trials (Janssens, B. et al., 2018; Schwindling et al., 2018) and five used an uncontrolled pre-post design (Bonwell et al., 2014; Forsell et al., 2011; Portella et al., 2015; Red and O'Neal, 2020; Sloane et al., 2013). All but one study (Portella et al., 2015) were performed in Europe (De Visschere et al., 2011; Forsell et al., 2011; Janssens, B. et al., 2018; Johansson et al., 2020; Overgaard et al., 2022b; Putten et al., 2013; Schwindling et al., 2018; Seleskog et al., 2018; Zenthöfer et al., 2016) or North America (Amerine et al., 2014; Bonwell et al., 2014; Le et al., 2012; Red and O'Neal, 2020; Sloane et al., 2013; Weintraub et al., 2018). Table 2 summarizes the basic characteristics of the studies and their outcome measurements.

**Table 2:** Study characteristics of the studies on improving oral health care (*n* = 16).

Study (Year)	Country	Design	Setting	Study population (n)	Implementation on ward by	OHC performed by	Selected outcome	Longest follow-up
Amerine et al., 2014	USA	RCT	LTC	Residents, intervention (58), control (20)	Registered dental hygienist (MSDH)/ Dental Hygiene Champion	CNA's	Oral Health Assessment Tool – 8 categories, including lips, tongue, gums and tissues, saliva, natural teeth, dentures, oral cleanliness and dental pain	8 weeks
Bonwell et al., 2014	USA	UBA	LTC	Nursing staff (88)	Periodontist, oral pathologist, pharmacist, dietitian, occupational therapist	Variety in disciplines, majority members of all levels of nursing care	Knowledge gained with questionnaire	Immediately after education
DeVisschere et al., 2011	Belgium	RCT	Nursing homes	14 NH's, residents, intervention (211), control (671 and 511)	Registered nurses as oral health coordinators, at least one nurse per ward	Nursing home staff, nursing assistants, nurse’ aides	Denture plaque Augsburger and Elahi/ Dental plaque by Silness and Loë	5 years
Forsell et al., 2011	Sweden	UBA	Single nursing home	Nursing staff (105)	Dental hygienist/ psychologist	Nurses, nurse assistants, nursing auxiliaries and nursing staff without formal education	Experiences of nursing staff (unpleasantness, resistance)	Unknown
Janssens et al., 2018	Belgium	CCT	Nursing homes	40 NH's, nursing staff, intervention (1888), control (521)	Oral health care team (oral health coordinator and at least one nurse or nurse aid per ward)	Nurses and nurses’ aides	Knowledge and attitude	Ranging from 13 – 18 months
Johansson et al., 2020	Sweden	RCT	Single nursing home	Nursing staff (48) Residents (58)	2 dental hygienists	Nurses, nurse assistants and registered nurses	Knowledge and attitude – Dental Coping Beliefs Scale (DCBS) Revised Oral Assessment Guide (ROAG) and Mucosal Plaque Score (MPS)	9 months
Le et al., 2012	Canada	RCT	Nursing homes	Nursing staff intervention (29), control (47)Residents intervention (41), control (39)	unknown	Nursing home support staff (not specified)	Oral care knowledge assessment tool, 20 items: 14 dichotomized true/false questions and six multiple-choice questions.Modified Plaque Index (PI) and Modified Gingival Index (GI)	6 months
Overgaard et al., 2022b	Denmark	RCT	Nursing homes	15 NH's, residents intervention (145), control (98)	Project dentist and dental practitioner	Nursing home staff (not specified) and residents themselves	Mucosal Plaque Score (MPS)	1 year
Portella et al., 2015	Brazil	UBA	Single nursing home	Residents (80)	Dental students and a professor	Nursing home staff (professional caregivers), majority nurse auxiliaries	Mucosal Plaque Score (MPS)	1 year
Red and O'Neal, 2020	USA	UBA	Single nursing home	Nursing staff (29) and residents (10)	Team including CNAs, RNs, a nurse practitioner, nurse scientists and a dentist	Nursing home staff, variety of training levels, majority CNA's, residents were partially assisted	Oral Care Questionnaire (knowledge and attitude) OHAT sum score	2 weeks
Schwindling et al., 2018	Germany	CCT	Nursing homes	14 NH's, residents, intervention (178), control (91)	One dentist	Professional nursing caregivers	Plaque Control Record (PCR), Gingival Bleeding Index (GBI), Community Periodontal Index of Treatment Needs (CPITN) and Denture Hygiene Index (DHI)	1 year
Seleskog et al., 2018	Sweden	RCT	Nursing homes	2 NH's, residents, intervention (15), control (22)	2 dental hygienists	Nursing staff (director of nursing, registered nurse and nursing assistants)	Revised Oral Assessment Guide (ROAG), dental plaque by Silness and Loë, Gingival Bleeding by Loë and Silness	3 months
Sloane et al., 2013	USA	UBA	Nursing homes	3 NH's, residents (97)	Dental hygienist and geriatric psychologist	CNA's	Plaque Index for Long-Term Care (PI-LTC), Gingival Index for Long-Term Care (GI-LTC)2, for dentures Denture Plaque Index (DPI)	8 weeks
Van der Putten, 2012	The Netherlands	RCT	Nursing homes	12 NH's, with sample of residents, intervention (177), control (166)	Dental hygienist supervisor, each ward had a nurse as oral health care organizer	Nursing home staff, nurses and nurse assistants	Denture plaque Augsburger and Elahi/ Dental plaque by Silness and Loë	6 months
Weintraub et al., 2018	USA	RCT	Nursing homes	14 NH's, residents, intervention (121), control (98)	Dementia specialist/ dental hygienist/ Dental Hygiene Champion	Nursing home staff; licensed nurses, registered nurses, CNA's	Plaque Index for Long-Term Care (PI-LTC), Gingival Index for Long-Term Care (GI-LTC)2, for dentures Denture Plaque Index (DPI)	2 years
Zenthöfer et al., 2016	Germany	RCT	Nursing homes	14 NH's, residents, intervention (144), control (75)	Dentists	Nursing home staff (not specified)	Plaque Control Record (PCR), Gingival Bleeding Index (GBI), Denture Hygiene Index (DHI) and Community Periodontal Index of Treatment Needs (CPITN)	6 months

RCT = randomized controlled trial, CCT = controlled clinical trial, UBA = uncontrolled before after, LTC: long term care; NH: nursing home, OHC: oral health care; MSDH = master of science in dental hygiene, CNA's = certified nursing assistants

The study settings were nursing homes, and two studies specifically mentioned that the setting was a long-term care facility for older people (Amerine et al., 2014; Bonwell et al., 2014). The included older people (residents) varied in number, from one pilot study including 37 residents of two nursing homes (Seleskog et al., 2018) to another study including 1393 participants from 14 nursing homes (De Visschere et al., 2011).

Three studies targeted only nursing staff's attitudes and/or knowledge (Bonwell et al., 2014; Forsell et al., 2011; Janssens, B. et al., 2018), whereas the other 13 studies (also) included residents’ oral health outcomes. Most studies that used the oral health of residents as an outcome reported measurements of dental or denture plaque using validated instruments, such as the Augsburger and Elahi denture plaque index (Augsburger and Elahi, 1982) or the Silness and Loë plaque index (Silness and Loe, 1964). The studies varied in the follow-up period from two weeks (Red and O'Neal, 2020) to five years (De Visschere et al., 2011). The implementation was performed by a dental professional in 14 of the 16 included studies. One study used registered nurses who were trained before implementation as oral health coordinators to implement an oral healthcare program in their wards (De Visschere et al., 2011) and another study did not report who supervised the implementation of video education (Le et al., 2012).

### 3.3 Content of strategies used and intensity of delivered strategies

The contents of the implementation strategies used in the studies are reported in Table 3. Appendix D provides a simplified overview of this table. The intensity of delivered strategies and the behavioral determinants that were addressed by these strategies are reported in Table 3. *Knowledge* was addressed in all studies in the current review. This was typically implemented as the transfer of information in (interactive) lectures with PowerPoint slides, discussions or question-and-answer sessions, and sometimes additional videos. Group discussions, question-and-answer sessions, and explanations were specifically mentioned in these studies. This was coded as BCT determinant “increase memory of understanding of information.”

**Table 3:** Overview of content of strategies, determinants addressed, and intensity of delivery in the 16 studies reviewed in 2024 and of 20 studies in 2013.

Study, year	BCT determinants addressed*									Content implementation strategies	Intensity of contacts
**Studies included in review 2024**											
	K	Aw	SI	A	SE	I	Ac	M	FB		
Amerine, 2014	X	X		X	X				X	Educational session, oral health protocol guidebook and discussion outlining relationships between oral health and systemic health, frequently seen oral health conditions, adequate oral hygiene care, and importance of regular oral assessment. The guidebook with information on provision of care subsequent to dental procedures (extractions, emergencies, etc.), daily oral health protocols, summary educational session. Dental Hygiene Champion answered questions and provided CNAs with professional hands-on dental hygiene support, encouragement, protocol compliance, advice for provision of oral health care to uncooperative residents and was an oral health advocate.	For 2 months monthly, 1×1 h Hands-on support for 8 weeks, 8 hrs a week
Bonwell, 2014	X	X			X					An Inter Professional Educational (IPE) approach training, PowerPoint and/or Keynote presentations and demonstrations, addressing interprofessional collaboration. Topics: oral-systemic relationship, oral pathology and instruction on extra and intra oral screening, oral health and pharmacology, overview of medications and fluoride use, poor oral health and food intake, instruction on oral hygiene care (techniques) and demonstration of adaptations and tools available to assist older people with different oral conditions.	5×45 minutes
DeVisschere, 2011	X				X	X			X	1. Oral health coordinators (OHC), responsible for implementation on wards, 2. theoretical and practical training of OHC, 3. Train the trainer, OHC trains nurses, nursing assistants or nurse’ aides (train the trainer), 4. oral assessment of new residents using assessment forms, 5. individualized oral hygiene plan and integration into daily care, to be performed by all care givers	1 h introduction director of institution half-day session for OHC
Forsell, 2011	X	X	X	X	X	X			X	Dental hygiene education program: 1. oral health assessment of the residents and instructions related to the residents’ individual needs for oral care. Hands-on training in toothbrushing technique using an electric toothbrush and providing electric toothbrushes to the residents and advice to use chlorhexidine; 2. discussion groups aiming to modify negative attitudes and perceptions of unpleasantness in relation to oral hygiene tasks and to encourage self-efficacy and a discussion about oral care for dementia patients, practical advice and recommendations of different oral hygiene products; 3. a theoretical lecture about dental hygiene, oral health, general health and well-being. The dental hygienist was available to the care staff	20-30 min per resident – staff members individual 60 min discussion 90 minute lecture Availability of dental hygienist on site 1 day a wk
Janssens, 2018	X	X	X	X	X	X			X	1. oral healthcare team, consisting of oral care aides at the different wards and one oral health coordinator, 2. education for managing director and for nurses and nurses’ aides, including hands-on training, 3. implementation of the Guideline for Older people in Long-term care Institutions (OGOLI) and the daily oral healthcare protocol 4. oral care aides (OCA) educated the nurses and nurses’ aides on their own wards (train-the-trainer concept). Oral health record for each resident aiming to facilitate behavior and to mobilize the social norm, 4. regular visits of mobile dental team to support nursing staff and to deliver preventive and curative oral health care for residents who could not access regular dental care.	Duration education unknown. Average days of visits of mobile dental team 6 days per NH (in an average period of 16 months).
Johansson et al., 2020	X	X		X	X	X			X	Oral health coaching program, two dental hygienists supported staff in observing, giving advice, answering questions, to be a coach and a resource in the daily care of residents, and to develop interprofessional relationships to the nursing staff. One workshop. Coaching in residents’ room and (practical) recommendations given to residents at yearly oral health assessment. DH recommended oral hygiene equipment and demonstrate oral care actions. DH gave feedback about how the staff performed oral care on the residents, on an individual level, DH provided information about causes and consequences of poor oral hygiene.	2 DH's 4 h/wk, for 3 months to support staff One workshop, duration unknown
Le et al., 2012	X	X								The oral care education program, “Mouth Care for Persons in Residential Care”: oral care video; covered the areas of common oral health conditions affecting residents of nursing homes, oral health promotion and disease prevention, daily mouth care provision, and oral care decision-making strategies to assist support staff in choosing the most appropriate oral care for residents	40 min video
Overgaard et al., 2022b	X	X			X	X			X	Lecture on oral health care (e-learning for control groups), oral healthcare plan was based on resident's level of functioning and oral hygiene status at each visit. Laminated version was placed in the resident's bathroom and included instructions on oral hygiene. Situated learning in oral care sessions, adjusted to the specific social interaction between the nursing home resident, nursing staff, and dental staff	One lecture/ e-learning In 6 months – first 2 mo weekly, month 3-4: every 2 wk, Mo 5-6: every 3 wk
Portella et al., 2015	X	X		X	X				X	An oral healthcare program, lecture and discussion including theory and practice of oral and body hygiene. Common risk factors for general and oral health, information on oral and dental diseases, prevention and oral hygiene instruction. A video on how to perform oral hygiene in a dependent individual. Practical demonstration/ training on models and dentures using tooth brushes and denture brushes. Image-based posters illustrating oral hygiene practices as guidelines: natural dentition, partial dentures and/ or complete dentures and specific equipment needed to perform oral hygiene care in these situations. Toothbrushes, denture brushes and toothpaste were supplied. Oral hygiene protocol was reinforced. Feedback was collected regarding difficulties during daily oral hygiene and staff received support and assistance.	2 hour lecture Video, duration unknown Reinforcement of oral hygiene protocol after 3 months, 3 times Assistance duration unknown
Red and O'Neal, 2020	X				X	X			X	Educational session and handouts; basic mouth care, denture care, oral care techniques, and standards of care. Caring for residents with disruptive behaviors and techniques to manage resistant care, demonstration on model dentures. Oral health protocol along with instructions on how to document daily oral care using a checklist. RN as oral health champion, to support staff. Free of charge oral care products for residents.	30-minute educational session, 18 minute presentation Oral health champion available
Schwindling et al., 2018	X	X			X				X	PowerPoint lecture (and handouts); oral problems and oral hygiene. A film with practical examples of oral healthcare measures and practical training on models about handling removable prostheses and brushing techniques for teeth and dentures. Dental care for volunteer residents by the staff members under supervision of the dentist. Provision of two devices for ultrasonic cleaning of prostheses per NH.	Duration of educational session and film unknown Supervised dental care not specified
Seleskog et al., 2018	X	X			X	X			X	1. Participation in staff meetings with the director of nursing and nursing staff: oral health care instructions and daily (individual) oral hygiene routines discussed. 2. Individualized theoretical and hands-on guidance and support for each resident once a week by DH's for 3 months and weekly 30 minute meeting to discuss oral hygiene procedures and issues. 3. Individualized written oral hygiene prescriptions for each resident for oral hygiene devices, procedures or products. 4. prescriptions placed in residents’ room with a signing sheet, to be completed each day.	2 hour staff meetings, 3 times in 3 months, 2 hours weekly hands on guidance of 2 DH's 15 min per resident and 30 minutes meeting with staff
Sloane et al., 2013	X	X		X	X				X	On-site training and consultation; seminars on oral pathology, dementia care, and individualized care planning plus skills training. The trainers provided care alongside the CNAs; a peer-to-peer approach as a team; training and supervision. Introduction of oral care protocols for natural teeth and dentures; providing of chlorhexidine and sodium fluoride paste. Strategies to reduce resistive behavior were addressed. Daily oral care record of care was provided.	Duration of seminars unknown, training and supervision daily for 2 wks, gradually decreased to few hours a wk for 8 wks
Van der Putten, 2012	X			X	X	X			X	1. 1.5-h informative oral presentation on guideline OGOLI, implementation of daily oral care protocol for managing staff and WOO's (Ward Oral Organizers), 2. Theoretical and practical training of WOOs: practical essentials of the guideline and oral care protocol. WOOs were trained in skills facilitating them to train nursing staff on their wards; train-the-trainer concept. 3. WOOs received all education materials (PowerPoint presentation, the OGOLI, daily oral care protocol and oral health care materials and products). 4. theoretical and practical education session by WOO's for all ward- nurses and nurse assistants: summary of the guideline was presented and all executive actions, such as tooth brushing, were taught and demonstrated with ward residents on site. WOO's encouraged and assisted staff in the daily delivery of oral care. WOOs were encouraged to organize repeating educational sessions for (new) staff.	6 months supervision; 6 wk monitoring visits, 1.5 h presentation for managing staff 2 h lecture, 3 h practical education of WOO's 1.5 h theory and practice by WOO's for nursing staff
Weintraub et al., 2018	X				X	X		X	X	Mouth Care Without a Battle (MCWB) program implementation with in-service presentation, training and instruction highlighting mouth care is healthcare (e.g., relates to pneumonia incidence); techniques and products to clean and protect the teeth, tongue, gums, and dentures (e.g., use of antimicrobial rinses); care provision in special situations (e.g., when teeth are broken or loose); and providing care to people who are resistant (e.g., singing, as a strategy to encourage residents to open their mouth) for nursing staff. Nursing assistant “champion” to support staff and to provide care to the residents who required the most time. Quality improvement techniques were used for monitoring and documentation activities, and reports of residents’ oral hygiene status	In-service presentation at baseline and after 12 months, duration unknown Monthly visits for 2 years, quarterly visits by investigators
Zenthöfer et al., 2016	X	X			X	X			X	Education program and training, a care movie and implementation of ultrasound baths for denture cleaning, with PowerPoint presentation. Topics: age-related changes and pathologies of the oral cavity and a standardized estimation tool of oral conditions, teeth brushing techniques, interdental space brushes and mouth rinses. Revised Oral Assessment Guide (ROAG), was implemented. Staff was trained in handling removable dentures using demonstration models and trained to use ultrasonic baths. Practical training in ROAG and hands on guidance in oral care practice with residents, dentist gave feedback and advice. CD-ROM and printed hand-outs of all lectures provided. Leading staff was trained as multipliers in communication training to their colleagues, incl exercises.	2 day program
**Studies included in review 2013**											
	K	Aw	SI	A	SE	I	Ac	M	FB		
Boczko, 2009	X									Power point with handouts and diagrams: definition of oral hygiene, elements of good oral care, identification of risk factors, the patient population and residents with behavior problems.	1×1 hour
Budtz, 2000	X				X	X			X	Interactive lecture with slide projections, followed by practical demonstration how to brush the teeth of dependent residents. Information on the etiology of caries, periodontal diseases, denture-induced lesions, basic principles of preventive measures in oral health care including denture wearing habits and dietary advice. Prophylactic treatment including scaling by dental hygienist and a recall system adapted to the patients’ needs, minimum 6 months.	1×45 min
DeVisschere, 2010	X				X				X	1. Project supervisor; 2. Oral health care team, including Ward Oral Organizers; 3. Supervision of implementation of the guideline. Train the trainer- concept: oral health team trains nurses, nurses (Ward Oral Organizers) train nurse aides and care aides. Free of charge oral health care products and materials.	1×1.5 hour
Fallon, 2006	X									5 stages: project development, interactive oral health education, Oral audits of patients with dementia, changes to oral health practice via care plans, critical reflection.	3×1 hour
Frenkel, 2001	X				X				X	Oral health care education session covering the role of plaque in oral disease, demonstrations of cleaning techniques and practice of these techniques on manikin heads and models, for dentures and natural teeth. Distribution of toothbrushes.	1×1 hour
Frenkel, 2002	X			X	X					Oral health care education session covering the role of plaque in oral disease, demonstrations of cleaning techniques and practice of these techniques, caregivers had an opportunity to discuss their feelings about oral health. Participant were given a worded booklet on oral healthcare and received a course attendance certificate.	1×1 hour
Isaksson et al., 2000	X					X				Oral health education program of 120 slides, video and compendium "Oral health care knowledge for nursing personnel", discussion and demonstration.	4×1 hour
Jäger et al., 2009					X					Oral health education program: theory concerning good oral health, consequences of bad oral health, definitions and treatments of oral health problems, demonstrations, attributes and cooperation with dentists.	1×1.5 hour and individual follow-up lessons
Kullberg, 2010	X				X				X	Theoretical lecture focusing on the association among dental hygiene and oral health, general health and well-being in elderly. Individual instructions to residents' contact persons. Hands-on training in tooth brushing technique and practical advice. Discussion groups about oral care for patients with dementia, with emphasis on research evidence on possible associations between oral health and general health in older people; modify negative attitudes and perceptions of unpleasantness of oral hygiene tasks; encourage nursing staff to contribute with own ideas to ensure them they were capable of finding solutions. Residents received an individual electric tooth brushes after education. Residents with own teeth were recommended to use Chlorhexidine (1 week every month, twice a day). Access of the dental hygienist for the care staff 1 day a week at the nursing home and all days by telephone.	1×30 min. + 1×60 min + 1×90 min.
MacEntee, 2007	X				X				X	Theoretical seminar. Access to approach the nurse educator for help and advice. Dental hygienist telephoned the nurse educator within 2 weeks of their first meeting to offer additional guidance or information. Nurse educator had telephonic access to the dental hygienist for advice on managing specific problems.	1×1 hour
Mojon, 1998	X				X				X	Interactive lecture with slide presentation providing information on the etiology of caries and periodontal pathologies, basic principles of oral health prevention and dietary advice. Practical demonstration on how to brush teeth of dependent residents. Prophylactic treatment provided by dental hygienists, experimental group, personal advice and recall program. Materials (toothbrushes etc.) were supplied.	1×45 min
Nicol et al., 2005	X		X		X					Training session: 30 min lecture. Followed by discussion of protocols and practical demonstrations. Local patients were invited to discuss their oral problems with the course participants. Encouraging to discuss encountered problems in providing oral care to patients.	1×1,5 hour
Paulsson, 1998	X									Oral health education program: 120 slides, video and compendium "Oral health care knowledge for nursing personnel", discussion and demonstration.	1×1 hour
Paulsson, 2001	X									Oral health education program: 120 slides, video and compendium "Oral health care knowledge for nursing personnel", discussion and demonstration.	1×1 hour
Pronych, 2010	X			X	X				X	Creation of an Oral Health Coordinator position at each site. Training by oral health coordinator consisted didactic training and job shadowing. Modified brushing protocols were offered. Credits towards relicensing.	1×1 hour
Reed et al., 2006	X				X					Power point presentation. Workshops included relevant issues to the relationships between medical and dental health and manifestations of disease. Hands-on presentations of oral health techniques with role-playing. Additional workshops provided problem solving and hands-on oral hygiene demonstrations with tooth models and live patients.	7 workshops
Samson, 2009	X	X			X				X	Oral health education program: teaching /motivation, group work based on discussions of actual patients, distribution of written information; production of picture-based procedure cards for each patient, constitutes an individual treatment plan; distribution of adequate appliances as toothbrushes and tooth paste; implementation of new routines on the ward, incl. an 'oral-care contact' person; regular measuring routines (follow-up/screening) and feedback on the residents' oral hygiene.	1×4 hours
Simons et al., 2000	X				X	X				Oral health training: demonstration with visualization of plaque, tooth brushing and denture cleaning techniques; practical involvement of the carers in cleaning each other's teeth, a video and information on diet, discussion; introduction of basic oral health assessment and individual oral care plans for all residents; training manual, box of samples and oral health aids, information leaflets and lists of places to obtain products.	1×1.5 hour
Wårdh et al., 2002a	X				X				X	Theoretical and practical education in oral health care. Supplemental the intervention group received support from the Oral Care Aide (Oral Care Aide attended the dental clinic for an educational program).	3×1 hour
Wårdh et al., 2002b	X				X				X	Oral health care training and Oral Care Aide (same as Wårdh et al., 2002a)	3×1 hour

*K = Knowledge, Aw = Awareness A = attitude, SI = Social Influence SE = Self-efficacy, I = intention, Ac = Action control, M = maintenance, FB = facilitation of behavior

*Awareness* was presented as the BCT's “risk communication” or “feedback on clinical outcomes.” Nursing staff were asked to complete an oral assessment of an older person as an example, after which feedback was provided on the outcomes. Risk communication was included in educational sessions, and the impact of poor oral hygiene and health was explained to the nursing staff. Feedback on clinical outcomes was often provided by dental care professionals who coached the nursing staff during or after oral assessments of nursing home residents.

*Social influence* as a strategy was used in only two studies (Forsell et al., 2011; Janssens, B. et al., 2018) and was implemented as “mobilizing the social norm.”

*Attitude* was targeted with praise and encouragement of the nursing staff and efforts to encourage them to actively participate (Amerine et al., 2014; Forsell et al., 2011; Janssens, B. et al., 2018; Johansson et al., 2020; Portella et al., 2015; Putten et al., 2013; Sloane et al., 2013). An additional method to improve attitude was allowing nursing staff to reevaluate clinical outcomes.

*Self-efficacy* was used often, mostly through the modeling and demonstration of oral healthcare techniques or guided practice. Self-efficacy in the current included studies was also enhanced using “reattributional training.” This was accomplished by attributing failure in performing oral care to the behavior of the older person and discussing different oral care techniques and skills that can be applied by the nursing staff.

*Intention* as strategy was used by specific goal setting or goal directed behavior, tailored oral care plans for older people, and social support (mostly from dental care staff)(De Visschere et al., 2011; Forsell et al., 2011; Janssens, B. et al., 2018; Johansson et al., 2020; Overgaard et al., 2022b; Putten et al., 2013; Red and O'Neal, 2020; Seleskog et al., 2018; Weintraub et al., 2018; Zenthöfer et al., 2016).

*Action control* is an implementation strategy that has not been used yet. In the BCT coding manual, “self-persuasion” or “use of cues” are the determinants of this strategy.

*Maintenance* was targeted in one study (Weintraub et al., 2018). We identified the quality improvement techniques, monitoring, and documentation carried out during the two years of the implementation of an oral care program to be a determinant of “maintenance.”

*Facilitation of behavior* was used in 14 of the 16 studies (Amerine et al., 2014; De Visschere et al., 2011; Forsell et al., 2011; Janssens, B. et al., 2018; Johansson et al., 2020; Overgaard et al., 2022b; Portella et al., 2015; Putten et al., 2013; Red and O'Neal, 2020; Schwindling et al., 2018; Seleskog et al., 2018; Sloane et al., 2013; Weintraub et al., 2018; Zenthöfer et al., 2016). Facilitation of behavior often included provision of materials (toothbrushes and other oral care materials) and personalized regime (daily oral care plan), and in the current review studies, this is also “continuous professional support.”

The intensity of the implementation strategies varied from a single educational video of 40 min (Le et al., 2012) to a two-day educational program (Zenthöfer et al., 2016) or an eight-week implementation, including daily training and supervision in the first two weeks (Sloane et al., 2013).

### 3.4 Effectiveness of strategies

In Table 4, the effectiveness of the different strategies is shown. Outcome measurements that were used to measure older people's oral health were “plaque or dental plaque,” “gingivitis,” or a combined oral health measurement (such as the Mucosal Plaque Score). In the 2013 review, “candidoses” were also found to be an outcome measure of the oral health of older people.

**Table 4:** Effectiveness of strategies targeting specific determinants of behavior change, in studies reviewed in 2024 (16 studies) and 2013 (18 studies).

Strategies	% studies with significant positive effects (n = studies addressing strategy)							
	Oral Health						Knowledge and/ or attitude of nursing staff	
***Strategies addressing at least one of these determinants:***	Dental Plaque / Denture Plaque		Gingivitis		Oral Health combined (e.g. Mucosal Plaque Score)			
	2024	2013	2024	2013	2024	2013	2024	2013
Knowledge	80 (10)	-	43 (7)	-	33 (6)	-	100 (6)	-
Provide general information	-	75 (12)	-	67 (6)	-	77 (13)	-	100 (6)
Increase memory	-	100 (4)	-	100 (2)	-	100 (4)	-	100 (3)
Awareness	86 (7)	100 (1)	33 (6)	0 (0)	20 (5)	100 (1)	100 (5)	0 (0)
Social Influence	0 (0)	100 (1)	0 (0)	0 (0)	0 (0)	0 (0)	100 (2)	0 (0)
Attitude	100 (4)	0 (0)	50 (2)	0 (0)	33 (3)	0 (0)	100 (3)	100 (1)
Self-efficacy	78 (9)	-	50 (6)	-	33 (6)	-	100 (5)	-
Modeling	-	67 (9)	-	75 (4)	-	70 (10)	-	100 (1)
Practice, guided practice	-	67 (3)	-	100 (1)	-	67 (3)	-	100 (2)
Intention	67 (6)	-	50 (4)	-	25 (4)	-	100 (4)	-
Develop OH schedule	-	67 (3)	-	0 (0)	-	75 (4)	-	0 (0)
Action control	0 (0)	0 (0)	0 (0)	0 (0)	0 (0)	0 (0)	0 (0)	0 (0)
Maintenance	100 (1)	0 (0)	100 (1)	0 (0)	0 (0)	0 (0)	0 (0)	0 (0)
Facilitation of behavior	78 (9)	-	50 (6)	-	67 (6)	-	100 (4)	0 (0)
Provide materials to facilitate behavior	-	80 (5)	-	100 (2)	-	83 (6)	-	-
Continuous professional support	-	80 (5)	-	50 (2)	-	80 (5)	-	-
Individualize regimen	-	50 (2)	-	0 (0)	-	50 (2)	-	-
***Strategies addressing at least a combination of these determinants :***								
Knowledge x Awareness	71 (7)	0 (0)	33 (6)	0 (0)	20 (5)	0 (0)	100 (5)	0 (0)
Knowledge x Intention	60 (5)	67 (3)	50 (4)	0 (0)	25 (4)	75 (4)	100 (4)	0 (0)
Knowledge x Self-efficacy	78 (9)	0 (0)	50 (6)	75 (4)	33 (6)	70 (10)	100 (5)	100 (1)
Knowledge x Self-efficacy x Facilitation of behavior	78 (9)	0 (0)	50 (6)	50 (2)	33 (6)	71 (7)	100 (4)	100 (1)

Legend: - this BCT was not specified further (as was done in 2013), to prevent reporting results multiple times within a BCT, 0 (0) means that this BCT was not found in the studies, on that specific outcome measurement.

Knowledge and/or attitudes were used to measure the effects on nursing staff. In one study, a validated instrument, the Dental Coping Beliefs Scale, was used (Johansson et al., 2020), and other knowledge or attitude outcomes were assessed using non-validated questionnaires or assessments.

The three studies addressing only knowledge and/or attitudes of nursing home staff showed statistically significant improvements in knowledge and/or attitudes (Bonwell et al., 2014; Forsell et al., 2011; Janssens, B. et al., 2018), whereas two of the three other studies addressing the oral health of residents together with knowledge or attitudes of nursing staff showed less pronounced results; two studies showed no sustained results on the oral health of residents (Johansson et al., 2020; Le et al., 2012) and another study found an increase in knowledge, but no statistically significant result for attitude (Red and O'Neal, 2020).

In ten studies the effects of implementation were quantified using dental or denture plaque scores. The results for the plaque levels of the residents were mostly positive and statistically significant (Amerine et al., 2014; De Visschere et al., 2011; Schwindling et al., 2018; Seleskog et al., 2018; Sloane et al., 2013; Weintraub et al., 2018; Zenthöfer et al., 2016). For gingivitis, this was not the case; Table 4 shows that there are seven studies measuring gingivitis, and only three studies showed a statistically significant positive effect of implementation of oral care on gingivitis (Sloane et al., 2013; Weintraub et al., 2018; Zenthöfer et al., 2016).

When focusing on the period of follow-up and effects, the effects on plaque levels are overall positive in the short term, but these effects are often not sustained, as shown in three studies with a follow-up period of nine months to five years(De Visschere et al., 2011; Johansson et al., 2020; Overgaard et al., 2022a). Shortly after the interventions, decreased plaque levels were reported; however, in later follow-up measurements, plaque levels were similar to baseline measurements.

Regarding the sizes of the study samples, six larger study populations were included (samples of multiple nursing homes, including up to 1888 nursing staff members or more than 150 older residents)(De Visschere et al., 2011; Janssens, B. et al., 2018; Overgaard et al., 2022b; Putten et al., 2013; Schwindling et al., 2018; Zenthöfer et al., 2016), but these studies varied in target population, outcome measurements, implementation strategies used, and effectiveness of oral care implementation. Therefore, no conclusions could be drawn from this aspect.

### 3.5 Commonly combined strategies and effectiveness

Two studies used seven different strategies to implement oral care (Forsell et al., 2011; Janssens, B. et al., 2018) and nine of the 16 studies in this review used five different implementation strategies (Amerine et al., 2014; De Visschere et al., 2011; Johansson et al., 2020; Portella et al., 2015; Putten et al., 2013; Seleskog et al., 2018; Sloane et al., 2013; Weintraub et al., 2018; Zenthöfer et al., 2016). The strategies that were often combined were knowledge, awareness, attitude, intention, self-efficacy, and facilitation of behavior, as shown in Table 3. Strategies such as awareness, attitude, and intention were addressed simultaneously. These strategies were also combined with other strategies (Table 4).

Again, knowledge, self-efficacy, and facilitation of behavior were most often combined (nine studies for plaque and six studies for gingivitis) and are reported in Table 4. The combined effect of these strategies on dental or denture plaque in nursing home residents was 78 % (seven out of nine studies), while this combination of strategies was 50 % effective on gingivitis (three out of six studies). In the current review, seven studies using the combination “knowledge” and “awareness” measured dental plaque, six studies using this combination measured gingivitis, five studies used a combined oral health instrument, and five studies used knowledge/attitudes of nursing staff as an outcome measurement. The effects of combined oral health measures such as mucosa, oral hygiene, and other dental aspects were reported, with one instrument not being exclusively positive. Depending on the combination of strategies used, their effects on oral health varied from 20 % (knowledge and awareness) to 33 % (knowledge and self-efficacy) shown in Table 4.

### 3.6 Meta-analyses of dental and denture plaque

**Fig. 2:**
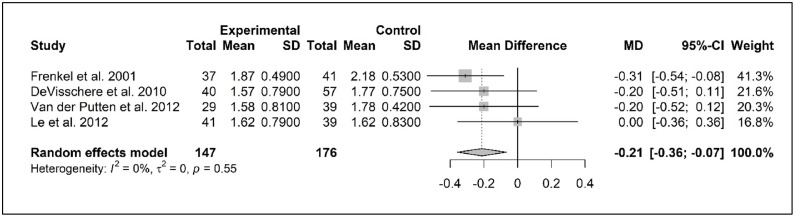
Forest plot dental plaque, at follow up 6 months.

Meta-analyses could be performed for two outcome measures: dental plaque and denture plaque. The four studies measuring dental plaque used the zero-to-three-point scale of Silness and Loë (Silness and Loe, 1964) and a follow-up time of six months (De Visschere et al., 2012; Frenkel, H. et al., 2001; Le et al., 2012; Putten et al., 2013). The sample sizes of the study groups were similar, ranging from 37 to 41 older residents. The pooled baseline data of the intervention and control groups are summarized in the forest plot given in Appendix E, showing a mean non-significant difference in plaque levels of .10 compared with the experimental group. The mean differences in the intervention groups of the different studies at baseline varied from 1.57 to 1.87 (De Visschere et al., 2012; Frenkel, H. et al., 2001; Le et al., 2012; Putten et al., 2013). In Fig. 2, a forest plot with the pooled follow-up data of the included studies is presented, showing a significant mean difference of -.21 (CI -.36; -.07, Cohen's *d -*.29). Given the range of zero to three on the scale, the size of this decrease is of minimal clinical value.

Test for overall effect *Z* = -2.85 *p<* 0.01

**Fig. 3:**
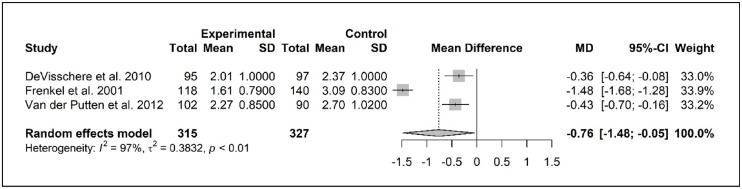
Forest plot denture plaque, at follow up 6 months.

The meta-analysis for denture plaque included three studies (De Visschere et al., 2012; Frenkel, H. et al., 2001; Putten et al., 2013), all of which used a zero-to-four-point scale developed by Augsburger and Elahi (Augsburger and Elahi, 1982). The study samples were larger than those used in the meta-analysis of dental plaque and varied from 95 to 118 older residents. The pooled baseline data of the intervention and control groups were compared in a forest plot (in Appendix E), showing a mean difference of denture plaque levels of -.05 in the experimental group as compared to the control group; the denture plaque score was slightly lower at baseline in the experimental group. The mean differences in the intervention groups of the different studies at baseline varied from 2.19 to 2.82 (De Visschere et al., 2012; Frenkel, H. et al., 2001; Putten et al., 2013). In Fig. 3, a forest plot with the pooled follow-up data of the included studies is presented, showing a significant mean difference of -.76 (CI -1.48; -.05, Cohen's *d* -.88) for denture plaque. Given the range of zero to four on the scale, this decrease is clinically relevant, as is also shown in Cohen's *d* of -.88.

Test for overall effect *Z* = -2.09 *p<* 0.05

The studies in both meta-analyses were the same, except for that by Le et al. (2012), which was only included in the dental plaque analysis. Three studies in the meta-analyses targeted “knowledge,” ”self-efficacy,” and “facilitation of behavior” (De Visschere et al., 2012; Frenkel, H. et al., 2001; Putten et al., 2013) and one study also enhanced ”awareness” and ”intention”(Putten et al., 2013), whereas one study only made use of ”knowledge” and “awareness” (Le et al., 2012).

### 3.7 Comparison of results of 2013 and 2024

First, as shown in Table 2, the quality of the studies included in 2024 was higher on average (67 %), compared to 47 % high-quality studies in 2013. Second, regarding the addressed strategies in the studies, it is shown in Table 3 that “awareness” was not often used as a strategy in the studies included in 2013. In the studies of the current review this was mostly operationalized as “feedback on clinical outcomes.” Social influence was addressed in one of the studies included in 2013 and again, only two studies used this strategy, as “mobilizing the social norm.” Self-efficacy was a commonly used strategy in the studies of 2013, but in the current included studies, self-efficacy was also enhanced using “reattributional training.” Again, “action control” is an implementation strategy that was not used in any of the included studies. In the review of 2013, ”facilitation of behavior” as a strategy was used in 50 % of the studies (Budtz-Jørgensen et al., 2000; De Visschere et al., 2012; Frenkel, Heather et al., 2002; Kullberg et al., 2010; MacEntee et al., 2007; Mojon et al., 1998; Pronych et al., 2010; Samson et al., 2009; Wårdh et al., 2002a, 2002b) mainly by providing toothbrushes, while in the current review studies, this is also “continuous professional support.”

Third, in the 2013 review, there was variety in the intensity of the delivered strategies and the duration of the programs. Fourth, concerning the combination of strategies, in the 2013 review, four studies used a single implementation strategy –”knowledge”– (Boczko et al., 2009; Fallon et al., 2006; Paulsson et al., 1998, 2001) whereas in the current review, all studies used multiple strategies to implement oral care. Compared to 2013, more strategies were addressed in different studies in the current review (Table 4). The combination of ”knowledge” and “awareness” was not present in the 2013 review.

Fifth, regarding the effectiveness of combinations that were used, studies measuring the combined oral health of older people were more often effective in the 2013 review. Seven studies (71 %) that addressed knowledge, self-efficacy, and facilitation of behavior showed significant positive effects and these effects were 33 % significantly positive in the current review (six studies).

Lastly, the outcome measurements for the effectiveness of the implementation of oral care in 2013 were almost equally divided between the oral health of older people and the nursing staff's knowledge and attitudes. In the current review, the oral health of older adults was assessed more often than nursing staff's knowledge/attitudes. This was more often studied with a measurement of plaque and/or gingivitis than with a combined oral health instrument, than found in the 2013 review.

## 4 Discussion

### 4.1 Main findings

In this systematic review, 16 papers published after the 2013 systematic review were of sufficient quality and, therefore, were included in the data extraction and meta-analysis. The current review confirmed what was revealed in 2013: the overall effects of strategies to implement oral care in nursing homes were mainly positive regarding the attitudes and knowledge of nursing home staff, yet their effects on the oral health of older people are not necessarily positive. However, the current review showed that more different strategies were used in the studies, and more combinations of strategies were enhanced. Although meta-analyses could be performed, we cannot confidently recommend the implementation strategies or combinations of strategies that are more or less effective in improving oral care in long-term care facilities. The studies in the meta-analyses used different combinations of implementation strategies; “knowledge,” “self-efficacy,” “facilitation of behavior,” and one study also used “awareness” and “intention.” The meta-analyses also showed that the study samples of dentate older people were smaller, as were the effects of oral care programs on oral hygiene, in comparison with older people with dentures.

### 4.2 Reflection on main findings

Plaque levels of nursing home residents were positively affected and improved, but long-term effects, for example, for gingival and combined oral health measures, were generally not statistically significant. In both reviews, the follow-up time of studies was mostly limited to 6 months; therefore, it is difficult to draw conclusions about the effects of implementations beyond this period. In the 2013 review, the effects were more positive, with percentages varying from 50 % to 100 % on combined oral health, compared to the current review (percentages varying from 20 % to 67 % on combined oral health). This may also reflect the nature of oral health problems and the selection of studies. To improve gingival health and reduce caries, dental treatment may be needed, yet we have only selected studies that included daily oral care by nursing staff. However, when daily oral care leads to behavioral changes that yield sufficient oral care over a longer period of time, this will also positively affect other aspects of oral health, such as the mucosa. Another explanation could be that barriers to behavioral change in nursing staff are present and of multiple origins: lack of time, uncooperative older people, lack of materials for oral care, lack of knowledge on how to perform oral care, and lack of collaboration between the dental and nursing staff (Gopalakrishnan et al., 2019; Göstemeyer et al., 2019; Hearn and Slack-Smith, 2015; Hoben et al., 2017). These barriers should be considered when implementing oral care improvement programs. It may not be realistic to expect an improvement in daily oral care when high workload and understaffing are experienced, leading to incomplete or nonexistent provision of oral care (Edfeldt et al., 2023; Mainz et al., 2024).

Another aspect to be considered is the relationship between self-efficacy and knowledge (Aro et al., 2021; Pihlajamäki et al., 2016). These two studies showed that self-efficacy increased after providing knowledge through education on oral health and hygiene practices. Nurses had no confidence in their ability to manage oral diseases (low self-efficacy), and it was suggested that there was a need to educate nursing staff (Pihlajamäki et al., 2016).

With the additional studies, we were able to perform a meta-analysis of two outcome measurements, dental and denture plaque, showing that the implementation of oral care in nursing homes significantly (and clinically) impacted denture plaque. Knowledge as a strategy was used in all studies in the meta-analysis, and this was combined with self-efficacy and the facilitation of behavior in three studies. Possible explanations have been reported for the more pronounced effects on denture plaque by other researchers: nurses often have more knowledge about denture brushing and may find it easier to clean dentures than to perform oral care for older people with natural teeth. Nursing staff are often uncertain about their provision of oral care in older people with natural teeth (Aro et al., 2018; Bellander et al., 2021). These researchers have also suggested that on-the-job education with practical training and guidance may lead to more positive outcomes in oral care for people with natural teeth. Implementing oral care programs that make use of inter-professional collaboration between nursing and dental staff, with a focus on improving the skills of nursing staff and targeting barriers to oral care in older people, can be promising, as shown in a qualitative study (Keboa et al., 2019).

In the current review, more high-quality studies were conducted than before 2013. This finding confirms the results of the 2013 review, showing that it is challenging to improve the oral health of older people in nursing homes, but that it is possible to conduct RCTs, resulting in high-quality evidence and making meta-analyses possible. More long-term follow-up studies are needed to determine whether oral care is maintained after initial implementation. However, we acknowledge that this is a difficult issue in older nursing home populations.

### 4.3 Limitations

Future research should use valid and reliable instruments to measure the oral health of older people and the knowledge, attitudes, and oral care behaviors of nursing staff. Unfortunately, the validity and reliability of knowledge and oral care behavior measurements are scarcely reported, which complicates the replication and comparison of the study results. In addition, there is no gold standard instrument or assessment to measure oral health and hygiene that can be used by dental care professionals (Bakker et al., 2024). The fact that multiple oral health and hygiene assessments are used in researching older people's status complicates the comparison of different study results. Therefore, it is recommended to use one or more existing instruments. Another limitation is that we only included a small number of studies; thus, reporting effectiveness as a percentage may have distorted the study report. We did so because this was also done in 2013, so that the study results could be directly compared.

Regarding the meta-analysis, currently no minimally important clinical differences have been described in the literature concerning dental and denture plaque. This limits the interpretation of the findings of these meta-analyses, which are based on the statistical effect size (Cohen's *d)* and may not necessarily represent the judgment of a clinical effect. However, the average denture plaque scores ranged from 1.61 to 2.27 at baseline with a mean difference decrease of .76; most older people in the samples had a score of 1 in the post measurements, which is “light” plaque, covering 25 % of the surfaces.

Additionally, in conducting systematic reviews, there is an issue of publication bias; “negative” research results are not (easily) published, and therefore underrepresented in systematic reviews and meta-analyses.

## 5 Conclusions

All studies in this review used knowledge as an implementation strategy, combined with strategies based on intention, awareness, self-efficacy, attitude, and facilitation of behavior. Implementation strategies positively affected the knowledge and attitudes of nursing staff, whereas the oral health of older people did not necessarily improve. Depending on the context, implementation strategies should be carefully selected to target barriers and mobilize facilitators experienced by the nursing home staff. Meta-analyses of plaque showed that oral care implementations are effective; for denture plaque, the effect size was large and thus may have more clinical value than for dental plaque. Further research using valid and reliable instruments is needed, with specific attention paid to older people with natural teeth.

## Registration information

The study was not registered.

## Review protocol

We used the same review protocol as the review protocol of 2013

## Funding statement

No funds for this research project were given.

## Data availability

Materials used in this review are to be found in the supplementary files or in the references provided. Further data and files are available at the first author on request.

## CRediT authorship contribution statement

**Lina F. Weening-Verbree:** Writing – review & editing, Writing – original draft, Visualization, Validation, Software, Project administration, Methodology, Investigation, Formal analysis, Data curation, Conceptualization. **Anouk Douma:** Writing – review & editing, Writing – original draft, Visualization, Validation, Supervision, Methodology, Investigation, Formal analysis, Data curation. **Cees P. van der Schans:** Writing – review & editing, Supervision, Methodology. **Getty J. Huisman-de Waal:** Writing – review & editing, Writing – original draft, Supervision, Methodology, Investigation, Formal analysis, Conceptualization. **Annemarie A. Schuller:** Writing – review & editing, Supervision, Methodology, Investigation. **Sytse U. Zuidema:** Writing – review & editing, Supervision, Methodology, Investigation. **Wim P. Krijnen:** Writing – review & editing, Writing – original draft, Visualization, Supervision, Software, Methodology, Investigation, Conceptualization. **Johannes S.M. Hobbelen:** Writing – review & editing, Writing – original draft, Supervision, Methodology, Investigation, Conceptualization.

## Declaration of competing interest

No conflict of interest has been declared by the author(s).

## Appendix Supplementary materials

Image, application 1

Image, application 2
