# Supplementary material for: Oral health care in older people in long-term care facilities: An updated systematic review and meta-analyses of implementation strategies
Source: Int J Nurs Stud Adv. 2024 Dec 31;8:100289. doi: 10.1016/j.ijnsa.2024.100289 (PMC11757228; doi:10.1016/j.ijnsa.2024.100289)
Supplement: Supplementary file 1 [file mmc1.docx]

# **Appendix A,** search terms September 2011 to June 2023

| **Database** | **Search terms** |
| --- | --- |
| Medline | ("Nursing"[Mesh] OR "Caregivers"[Mesh] OR "Nursing Homes"[Mesh] OR "nursing"[TW]) AND ("Oral Hygiene"[Mesh] OR "Oral Health"[Mesh] OR "Health Education, Dental"[Mesh] OR oral hygiene[TI] OR oral health[TI]) AND ("Aged"[Mesh] OR "elderly") |
| Cinahl | (MH "Aged+") AND ((MH "Oral Hygiene+") OR (MH "Oral Health") OR "oral health") AND ((MH "Nursing Care+") OR (MH "Nursing Assistants") OR (MH "Nurses+") OR (MH "Nursing Home Personnel") OR (MH "Nursing Homes+")) |
| Cochrane | ((MeSH descriptor Aged explode all trees OR MeSH descriptor Health Services for the Aged explode all trees OR elder*) AND ( MeSH descriptor Oral Health explode all trees OR MeSH descriptor Oral Hygiene explode all trees) AND (MeSH descriptor Nursing explode all trees OR MeSH descriptor Nursing Care explode all trees OR nurs*)) |

# **Appendix B:** Methodological quality rating (adapted for Anderson & Sharpe 1991)

|  | **Rating** |
| --- | --- |
| **Design of study or assignment** |  |
| Experimental: RCT, random allocation; CCT, quasi-random allocation; ITS, three data collection points before and after the intervention | 1 |
| Quasi-experimental: CBA, comparable control sites | 1 |
| Quasi-experimental: non-equivalent control sites | 0 |
| Single group before-after test with baseline measurement | 0 |
| **Content** |  |
| Intervention is clearly described | 1 |
| **Sample size** |  |
| Described and justified. An *n* per group sufficient to detect a significant effect (p < 0.05) with a power of 0.80 or reported calculation of power | 1 |
| **Validity and reliability of instruments** |  |
| ***Primary outcome**** |  |
| Observing and measuring ≥ 2 aspects (e.g. Plaque (DI-S, PI, plaque disclosing solution), Gingivitis (GI or GBI), Candidosis or other oral hygiene aspects) | 2 |
| Observing and measuring 1 aspect (e.g. Plaque (DI-S, PI, plaque disclosing solution), Gingivitis (GI or GBI), Candidosis or other oral hygiene aspects) | 1 |
| Observing none of the mentioned aspects | 0 |
| ***Secondary outcome*** |  |
| Objective questionnaire, Internal Consistency Reliability and content validity described and *r >* 0.80 | 2 |
| Objective questionnaire, Internal Consistency Reliability and content validity not described or *r <* 0.80 | 1 |
| Self-evaluation | 0 |
| **Test statistics** |  |
| Test statistics are described | 1 |
| **Significance** |  |
| *p value or confidence interval is given* | 1 |

******when primary outcome AND secondary outcome 🡪 only score primary outcome*

CBA = controlled before-and-after study, CCT = controlled clinical rating, ITS = interrupted time series

**Appendix C:** Overview of terms and definitions from the Coding Manual for Behavioural Change Techniques

**Knowledge**

- Provide general information: *educational sessions or educational materials*
- Increase memory of understanding of information: *group discussion, answering questions, clarification*

**Awareness**

- Risk communication: *information about risks of non adherence or inadequate OH (infections, diseases)*
- Self-monitoring of behavior: *professional keeps a record of specified behaviors (notes, diary)*
- Self-report of behavior: *professional is asked to self report behavior at least concerning the last 3 days*
- Electronic monitoring of behavior: *professional is asked to monitor OH behavior using an electronic monitoring device*
- Reflective listening: *direct feedback of cognitions and emotions during 1-on-1 communication with the professional intended to increase awareness of ideas, reasoning and emotions*
- Delayed feedback of behavior: *overview of recorded OH behavior*
- Direct feedback of behavior: *using a system to make professionals aware of their OH behavior soon after planned execution*
- Feedback of clinical outcomes: *overview of plaque, gingivitis, candidoses and other oral hygiene aspects*

**Social Influence**

- Provide information about peer behavior: *information about peers’ opinion of correct OH*
- Provide opportunities for social comparison*: group session with peers in which discussion and social comparison of OH behavior can occur*
- Mobilize social norm: *exposing professionals to the social norm of important others (not peers) such as family members or opinion leaders*

**Attitude**

- Reevaluation of outcomes, self-evaluation: *comparison of desired behavior with actual behavior, and reflections of ambivalence*
- Persuasive communication: *strengthen positive beliefs about the outcome of proper OH*
- Reinforcement on behavioral progress: *praise, encouragement, of material rewards*
- Reinforcement on motivational progress: *praise, affirming remarks, efforts to actively participate in the intervention program*

**Self-efficacy**

- Modelling: *use of a role model, demonstration of OH behavior and techniques in group of class*
- Verbal persuasion: *messages designed to strengthen efficacy/control beliefs about the way of performing correct OH*
- Guided practice: *rehearsing the OH skills or behavior and providing of feedback by the trainer*
- Plan coping responses: *determination of potential barriers and ways to overcome these.*
- Set graded tasks, goal setting: *achievement of difficult/complex goals with simple steps*
- Reattribution training, external attribution of failure: *attribute failure to an external but controllable factor so that the professional remains confident to attempt OH behavior in the future*

**Intention**

- General intention formation: *formulating a general behavior goal or the desired outcome of OH*
- Develop schedule concerning OH: *a written (time)schedule or tailored plan*
- Specific goal setting: *planning what the professional will do including a definition of the goal-directed behavior to improve OH*
- Review of general of specific goals: *reconsideration of previously set goals and the attempt to act on those goals*
- Agree behavioral contract: *contract or commitment to formulated goals of OH behavior*
- Use of social support: *social or emotional support provided by others to change OH behavior*

**Action control**

- Use of cues: *reminders*
- Self-persuasion: *self-motivating strategies, self-talks to increase motivation and confidence*

**Maintenance**

- Formulate goals for maintenance of behavior: *planning and/or intention on maintenance of OH behavior after change occurred*
- Relapse prevention: *following behavioral change, plan coping responses to (long-term) maintenance of OH behavior; prompt the professional to determine barriers and ways to overcome these*

**Facilitation of behavior**

- Provide materials to facilitate behavior: *supportive materials are provided to the professionals or patients*
- Continuous professional support: *services after the major part of the behavior change intervention has completed. Such as telephone calls, oral healthcare team or oral healthcare coordinator*
- Individualize regimen: *oral healthcare is explicitly tailored to the needs of the patient*
- Cope with side-effects: *physician informs for side-effects and takes steps to deal with it*
- Reduce environmental barriers: *reducing problems that compete for attention with the OH behavior*

# **Appendix D** provides detailed information on quality assessment and a simplified overview of Table 3, strategies used in implementation.

*Quality assessment for 18 studies assessed in 2024*

| **Author, year** | **Design** | **Strategy clearly described** | **Sample size described & justified** | **Validity & reliability of instruments** | | **Test statistics described** | **P-value or CI given** | **Quality rating^*^** |
| --- | --- | --- | --- | --- | --- | --- | --- | --- |
|  |  |  |  | Primary outcome | Secondary outcome^**^ |  |  |  |
| 1. Amerine, 2014 | 1 | 1 | 0 | 2 | (0) | 1 | 1 | 6 |
| 1. Bonwell, 2014 | 0 | 1 | 0 | 0 | (1) | 1 | 1 | 4 |
| 1. DeVisschere, 2011 | 1 | 1 | 0 | 2 | (0) | 1 | 1 | 6 |
| 1. Forsell, 2011 | 0 | 1 | 0 | 0 | (1) | 1 | 1 | 4 |
| 1. Janssens, 2018 | 1 | 1 | 1 | 0 | (1) | 1 | 1 | 6 |
| 1. Johansson, 2020 | 1 | 1 | 0 | 2 | (2)** | 1 | 1 | 6 |
| Le, 2012 | 1 | 1 | 1 | 2 | (1) | 1 | 1 | 7 |
| 1. McConnell, 2018 | 0 | 1 | 0 | 2 | (0) | 0 | 0 | 3*** |
| 1. Overgaard, 2021 | 1 | 1 | 1 | 2 | (0) | 1 | 1 | 7 |
| 1. Portella, 2013 | 0 | 1 | 0 | 2 | (0) | 1 | 1 | 5 |
| 1. Red, 2020 | 0 | 1 | 0 | 2 | (1) | 1 | 1 | 5 |
| 1. Schwindling, 2018 | 1 | 1 | 0 | 2 | (0) | 1 | 1 | 6 |
| 1. Seleskog, 2018 | 1 | 1 | 0 | 2 | (0) | 1 | 1 | 6 |
| 1. Sloane, 2013 | 0 | 1 | 0 | 2 | (0) | 1 | 1 | 6 |
| 1. Van der Putten, 2012 | 1 | 1 | 1 | 2 | (0) | 1 | 1 | 7 |
| 1. Volk, 2019 | 0 | 1 | 0 | 1 | (0) | 0 | 0 | 2*** |
| 1. Weintraub, 2018 | 1 | 1 | 0 | 2 | (0) | 1 | 1 | 6 |
| 1. Zenthöfer, 2016 | 1 | 1 | 0 | 2 | (0) | 1 | 1 | 6 |

* 3-5 moderate quality; 6-7 high quality

** secondary outcome only counted in absence of primary outcome

*** this study was excluded because no statistical analysis was described

*Quality assessment for 21 studies assessed in 2013*

| **Author, year** | **Design** | **Strategy clearly described** | **Sample size described & justified** | **Validity & reliability of instruments** | | **Test statistics described** | **P-value or CI given** | **Quality rate^*^** |
| --- | --- | --- | --- | --- | --- | --- | --- | --- |
|  |  |  |  | Primary outcome | Secondary outcome^**^ |  |  |  |
| 1. Boczko et al. 2009 | 0 | 1 | 0 | 2 | (1) | 1 | 1 | 5 |
| 1. Budtz et al. 2000 | 1 | 1 | 0 | 2 | (1) | 1 | 1 | 6 |
| 1. DeVisschere et al. 2010 | 1 | 1 | 1 | 1 | (1) | 1 | 1 | 6 |
| 1. Fallon et al. 2006 | 1 | 1 | 0 | 2 | (1) | 1 | 1 | 6 |
| 1. Frenkel et al. 2001 | 1 | 1 | 1 | 2 | (1) | 1 | 1 | 7 |
| 1. Frenkel et al. 2002 | 1 | 1 | 1 | 1 | (1) | 1 | 1 | 6 |
| 1. Isaksson et al. 2000 | 1 | 1 | 0 | 2 | (1) | 1 | 1 | 6 |
| 1. Jäger et al. 2009 | 0 | 1 | 0 | 2 | (1) | 1 | 1 | 5 |
| 1. Kullberg et al. 2010 | 0 | 1 | 0 | 2 | (1) | 1 | 1 | 5 |
| 1. MacEntee et al. 2007 | 1 | 1 | 1 | 2 | (1) | 1 | 1 | 7 |
| 1. Mojon et al. 1998 | 1 | 1 | 0 | 2 | (1) | 1 | 1 | 6 |
| 1. Nicol et al. 2005 | 1 | 1 | 0 | 2 | (1) | 1 | 1 | 6 |
| 1. Paulsson et al. 1998 | 0 | 1 |  | 2 | (1) | 1 | 1 | 5 |
| 1. Paulsson et al. 2001 | 0 | 1 | 0 | 2 | (1) | 1 | 1 | 5 |
| 1. Pronych et al. 2010 | 0 | 1 | 0 | 1 | (1) | 1 | 1 | 4 |
| 1. Reed et al. 2006 | 0 | 1 | 0 | 1 | (1) | 1 | 1 | 4 |
| 1. Rivett 2006 | 0 | 0 | 1 | 0 | (0) | 1 | 1 | 3*** |
| 1. Samson et al. 2009 | 0 | 1 | 0 | 2 | (1) | 1 | 1 | 5 |
| 1. Simons et al. 2000 | 1 | 1 | 0 | 2 | (1) | 1 | 1 | 6 |
| 1. Wardh et al. 2002A | 1 | 1 | 0 | 1 | (1) | 1 | 1 | 5 |
| 1. Wardh et al. 2002B | 1 | 0 | 0 | 2 | (1) | 1 | 1 | 5 |

* 3-5 moderate quality; 6-8 high quality

** secondary outcome only counted in absence of primary outcome

*** this study was excluded because the instruments used were not valid and reliable.

*Simplified overview of determinants addressed in the studies reviewed in 2024 and 2013.*

*K = Knowledge, Aw = Awareness A = attitude, SI = Social Influence SE = Self-efficacy, I = intention, Ac = Action control, M = maintenance, FB = facilitation of behavior

| **Studies included in 2023** | | **BCT determinants addressed*** | | | | | | | | |  | **Studies included in 2013** | **BCT determinants addressed*** | | | | | | | | |
| --- | --- | --- | --- | --- | --- | --- | --- | --- | --- | --- | --- | --- | --- | --- | --- | --- | --- | --- | --- | --- | --- |
| **Author, year** | K | | Aw | SI | A | SE | I | Ac | M | FB |  | **Author, year** | K | Aw | SI | A | SE | I | Ac | M | FB |
| Amerine, 2014 | X | | X |  | X | X |  |  |  | X |  | Boczko, 2009 | X |  |  |  |  |  |  |  |  |
| Bonwell, 2014 | X | | X |  |  | X |  |  |  |  |  | Budtz, 2000 | X |  |  |  | X | X |  |  | X |
| DeVisschere, 2011 | X | |  |  |  | X | X |  |  | X |  | DeVisschere, 2010 | X |  |  |  | X |  |  |  | X |
| Forsell, 2011 | X | | X | X | X | X | X |  |  | X |  | Fallon, 2006 | X |  |  |  |  |  |  |  |  |
| Janssens, 2018 | X | | X | X | X | X | X |  |  | X |  | Frenkel, 2001 | X |  |  |  | X |  |  |  | X |
| Johansson, 2020 | X | | X |  | X | X | X |  |  | X |  | Frenkel, 2002 | X |  |  | X | X |  |  |  |  |
| Le, 2012 | X | | X |  |  |  |  |  |  |  |  | Isaksson, 2000 | X |  |  |  |  | X |  |  |  |
| Overgaard, 2021 | X | | X |  |  | X | X |  |  | X |  | Jäger, 2009 |  |  |  |  | X |  |  |  |  |
| Portella, 2013 | X | | X |  | X | X |  |  |  | X |  | Kullberg, 2010 | X |  |  |  | X |  |  |  | X |
| Red, 2020 | X | |  |  |  | X | X |  |  | X |  | MacEntee, 2007 | X |  |  |  | X |  |  |  | X |
| Schwindling, 2018 | X | | X |  |  | X |  |  |  | X |  | Mojon, 1998 | X |  |  |  | X |  |  |  | X |
| Seleskog, 2018 | X | | X |  |  | X | X |  |  | X |  | Nicol, 2005 | X |  | X |  | X |  |  |  |  |
| Sloane, 2013 | X | | X |  | X | X |  |  |  | X |  | Paulsson, 1998 | X |  |  |  |  |  |  |  |  |
| Van der Putten, 2012 | X | |  |  | X | X | X |  |  | X |  | Paulsson, 2001 | X |  |  |  |  |  |  |  |  |
| Weintraub, 2018 | X | |  |  |  | X | X |  | X | X |  | Pronych, 2010 | X |  |  | X | X |  |  |  | X |
| Zenthöfer, 2016 | X | | X |  |  | X | X |  |  | X |  | Reed, 2006 | X |  |  |  | X |  |  |  |  |
|  |  | |  |  |  |  |  |  |  |  |  | Samson, 2009 | X | X |  |  | X |  |  |  | X |
|  |  | |  |  |  |  |  |  |  |  |  | Simons, 2000 | X |  |  |  | X | X |  |  |  |
|  |  | |  |  |  |  |  |  |  |  |  | Wardh, 2002A | X |  |  |  | X |  |  |  | X |
|  |  | |  |  |  |  |  |  |  |  |  | Wardh, 2002B | X |  |  |  | X |  |  |  | X |

# **Appendix E** Forest plots dental and denture plaque at baseline

Forest plot dental plaque at baseline.


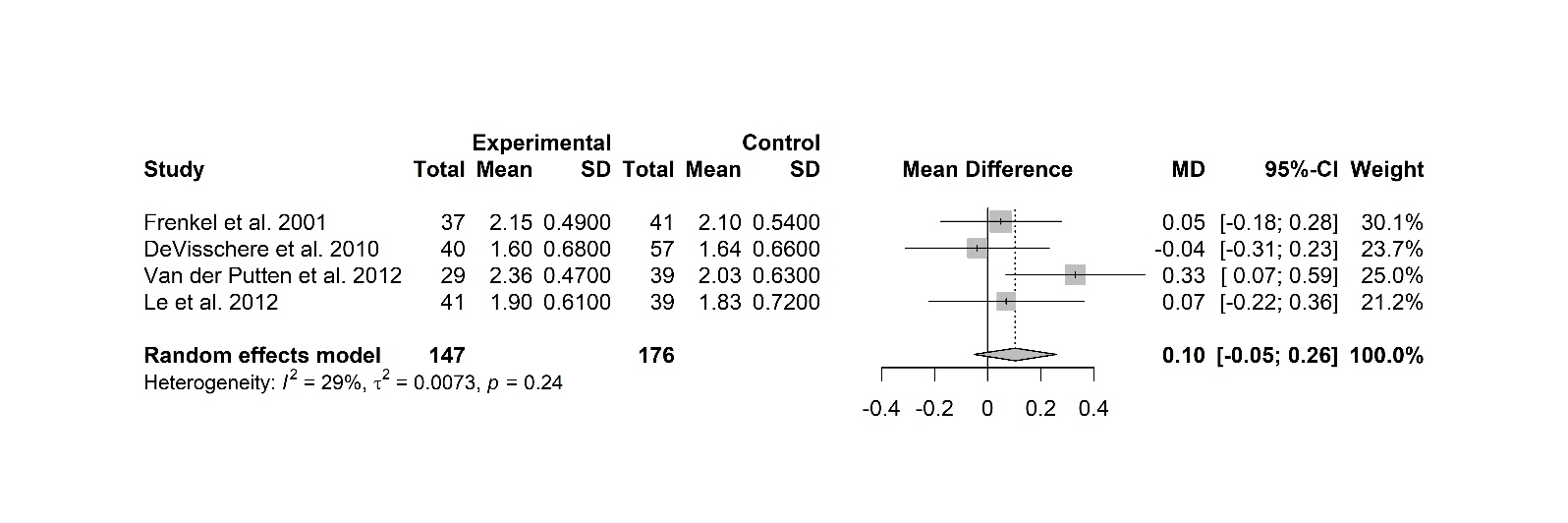


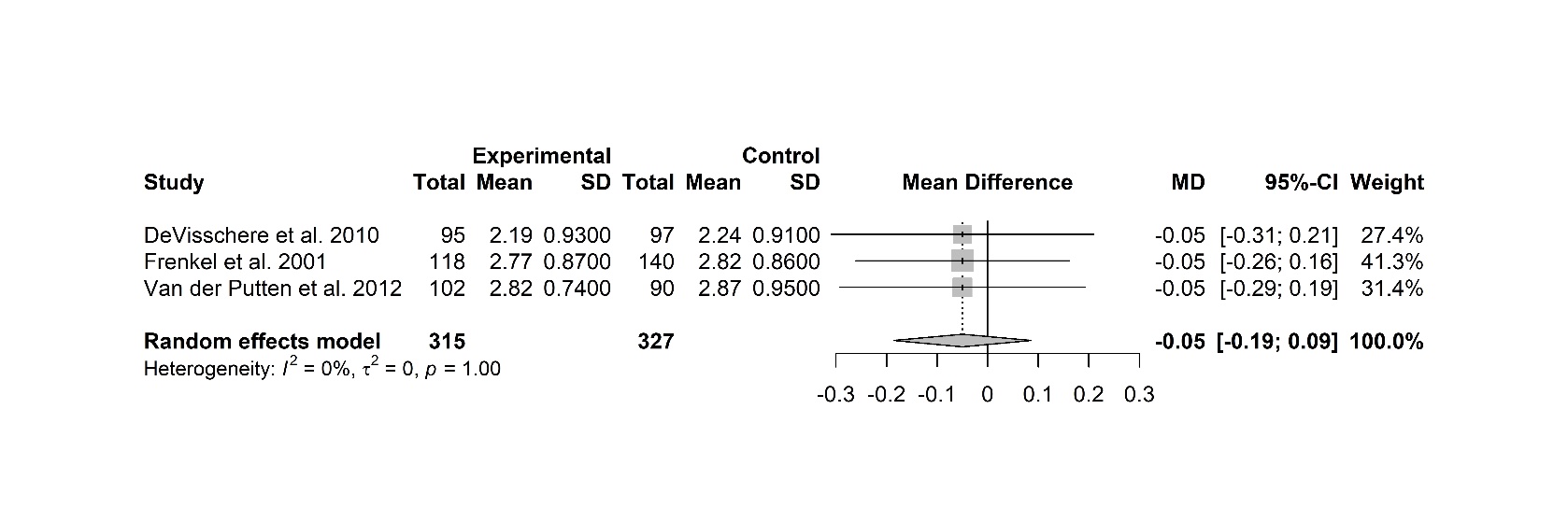
Forest plot denture plaque at baseline.
