# Supplementary material for: Oral health care in older people in long-term care facilities: An updated systematic review and meta-analyses of implementation strategies
Source: Int J Nurs Stud Adv. 2024 Dec 31;8:100289. doi: 10.1016/j.ijnsa.2024.100289 (PMC11757228; doi:10.1016/j.ijnsa.2024.100289)
Supplement: Supplementary file 2 [file mmc2.pdf]

ELSEVIER

Certificate of Elsevier Language Editing Services

The following article was edited by Elsevier Language Editing Services:

Oral health care in older people in long-term care facilities: an updated systematic review and meta-analyses of implementation strategies

Ordered by:

Linet Weening-Verbree

Estimated Delivery date:

2024-12-23

Order reference:

ASLESTD1089453
